# NKG2C^+^CD57^+^ Natural Killer Cell Expansion Parallels Cytomegalovirus-Specific CD8^+^ T Cell Evolution towards Senescence

**DOI:** 10.1155/2016/7470124

**Published:** 2016-05-29

**Authors:** John Heath, Nicholas Newhook, Emilie Comeau, Maureen Gallant, Neva Fudge, Michael Grant

**Affiliations:** Immunology and Infectious Diseases Program, Division of BioMedical Sciences, Faculty of Medicine, Memorial University of Newfoundland, St. John's, NL, Canada A1B 3V6

## Abstract

*Objective*. Measuring NKG2C^+^CD57^+^ natural killer (NK) cell expansion to investigate NK responses against human cytomegalovirus (HCMV) and assessing relationships with adaptive immunity against HCMV.* Methods*. Expansion of NKG2C^+^CD57^+^ NK was measured in peripheral blood mononuclear cells (PBMC) from groups distinguished by HCMV and human immunodeficiency virus (HIV) infection status. Anti-HCMV antibody levels against HCMV-infected MRC-5 cell lysate were assessed by ELISA and HCMV-specific CD8^+^ T cell responses characterized by intracellular flow cytometry following PBMC stimulation with immunodominant HCMV peptides.* Results*. Median NK, antibody, and CD8^+^ T cell responses against HCMV were significantly greater in the HCMV/HIV coinfected group than the group infected with CMV alone. The fraction of CMV-specific CD8^+^ T cells expressing CD28 correlated inversely with NKG2C^+^CD57^+^ NK expansion in HIV infection.* Conclusion*. Our data reveal no significant direct relationships between NK and adaptive immunity against HCMV. However, stronger NK and adaptive immune responses against HCMV and an inverse correlation between NKG2C^+^CD57^+^ NK expansion and proliferative reserve of HCMV-specific CD8^+^ T cells, as signified by CD28 expression, indicate parallel evolution of NK and T cell responses against HCMV in HIV infection. Similar aspects of chronic HCMV infection may drive both NK and CD8^+^ T cell memory inflation.

## 1. Introduction

Innate immune cells are cells that participate in immune defense against microbes but lack the clonotypic receptors responsible for diverse antigen-specific recognition and immune memory. These cells have conventionally been viewed as having a constitutive distribution and being incapable of selective adaptation towards secondary encounters with the same microbe. However, the line between innate and adaptive immunity based on clonotypic receptors is blurred by *γδ* T cells, natural killer T (NKT) cells, and B1B cells with strict partitioning further challenged by the behaviour of innate lymphoid cell (ILC) subsets, most notably that of NK cells. The “missing self” hypothesis of NK cell regulation was introduced 25 years ago, but the molecular details and a much deeper appreciation of the specialized degree of NK “education” towards self-tolerance and subsequent regulation of their effector functions have emerged over the last decade [1–3]. This has solidified the concept of substantial NK diversity based on subdivision into distinct subsets functionally specified by expression of a highly variable constellation of inhibitory and activating receptors.

The first illustration of NK specificity and memory involved NK from C57BL/6 mice shown to directly recognize a murine cytomegalovirus (MCMV) glycoprotein (m157) through activating receptor Ly49h [4, 5]. Expression of this receptor confers relative resistance of C57BL/6 mice to MCMV, primary infection with MCMV drives selective expansion of NK expressing Ly49h, and an expanded population of Ly49h^+^ NK that responds more effectively upon secondary MCMV exposure persists following primary infection [6–9]. This illustrates a clear exception to the rule that all immune memory depends on clonotypic receptors. Memory NK responses in the form of antigen-specific contact hypersensitivity mediated by murine NK that traffic to and from the liver have also been reported, but no mechanism for this specificity has been established [10]. In human studies, associations between killer cell immunoglobulin-like receptor (KIR) expression, human class I histocompatibility-linked antigen (HLA) genotype, and outcomes of viral infection suggest that particular subsets of NK cells have greater antiviral activity depending on the presence or absence of corresponding class I HLA ligands [11–13]. Expansion of NK expressing particular KIR occurs during acute human immunodeficiency virus (HIV) infection and there is evidence for NK escape mutations in chronic HIV infection [14, 15]. Recent studies show specific recognition of target cells pulsed with viral peptides or proteins by NK from peripheral blood of infected humans and by NK from the spleen and liver of simian immunodeficiency virus (SIV) or SHIV-infected macaques [16, 17]. Thus, there is growing evidence that subsets of NK mediate selective immunological responses.

Human (H)CMV infection leaves a distinct imprint on the NK repertoire involving expansion and accumulation of cells expressing the activating receptor NKG2C and maturation marker CD57 [18]. These cells also tend to lack expression of NKG2A, while expressing activating KIR and those inhibitory KIR with corresponding class I HLA ligands present [19–21]. Expression of NKp30 and NKp46 is reduced on these NK, which is reflected in lesser natural cytotoxicity; however, antibody-dependent activation of NKG2C^+^CD57^+^ NK is enhanced relative to other NK [18, 22]. A majority of the NKG2C^+^CD57^+^ NK do not express Fc*ε*R1*γ* or the signaling kinase SYK, suggesting an alternate form of intracellular signaling in association with antibody-mediated CD16 engagement [23–26].

Although there is no direct evidence that NKG2C mediates specific recognition of HCMV or HCMV-infected cells, expression of CD57 and NKG2C clearly demarcates an NK subset selectively expanded following exposure to HCMV [14–18]. Therefore, CD57^+^NKG2C^+^ NK frequency serves to measure NK responses against HCMV. As the relationship between NK and adaptive immunity against HCMV is largely unknown and likely to be important, we chose to investigate this relationship by comparing B and T cell responses against HCMV across a broad range of CD57^+^NKG2C^+^ NK frequencies, excluding data from NKG2C^null^ individuals. We determined the frequency of CD57^+^NKG2C^+^ NK, measured relative antibody levels against CMV-infected cell lysate, and characterized CD8^+^ T cell responses against immunodominant CMV proteins in over 200 individuals. A large group of HIV-infected individuals were incorporated into the study because exaggerated responses against HCMV in this population produce a broader range of responses for comparison [27, 28].

## 2. Materials and Methods

### 2.1. Study Subjects

Non-HIV-infected individuals were recruited from healthy Memorial University of Newfoundland Faculty of Medicine personnel. Individuals infected with HIV were recruited through the Newfoundland and Labrador Provincial HIV Clinic, St. John's, NL, Canada. All HIV-infected individuals were in the chronic stage of infection and most were receiving combination antiretroviral therapy at times of testing. Ethical approval for this study was granted by the Newfoundland and Labrador Health Ethics Research Authority and all participants provided informed consent for blood collection and immunological studies. Blood was collected by forearm venipuncture into acid-citrate-dextrose containing vacutainers and plasma for CMV antibody testing was collected by centrifuging whole blood at 400 ×g for 10 min, removing the upper acellular phase and immediately storing small aliquots at −80°C until testing. Peripheral blood mononuclear cells (PBMC) were isolated by Ficoll-Hypaque (GE Healthcare Biosciences, Mississauga, ON, Canada) density gradient centrifugation and suspended in lymphocyte medium consisting of RPMI 1640 supplemented with 10% fetal calf serum (FCS), 100 *μ*g/mL streptomycin, 100 IU/mL penicillin, 2 mM L-glutamine, 10 mM HEPES buffer, and 2 × 10^−5 ^M 2-mercaptoethanol (all from Invitrogen, Carlsbad, CA, USA), with FCS increased to 20% and dimethyl sulfoxide (Sigma-Aldrich, St. Louis, MO, USA) added to 10% for preservation in liquid N_2_ until testing.

### 2.2. Assessment of CD57^+^NKG2C^+^ NK Frequency

The percentage of NK in fresh PBMC expressing NKG2C and CD57 was assessed by multiparametric flow cytometry. Lymphocytes were identified by forward and side scatter, T cells excluded using anti-CD3^*∗*^peridinin chlorophyll protein (perCP) from Miltenyi Biotec, San Diego CA, USA (clone BW264/56), and NK cells identified with anti-CD56^*∗*^fluorescein isothiocyanate (FITC) from Ebioscience, San Diego, CA, USA (clone MEM188). Anti-NKG2C^*∗*^allophycocyanin (APC) from R&D Systems, Minneapolis, MN, USA (clone 134591), and anti-CD57^*∗*^phycoerythrin (PE), from Biolegend, San Diego CA, USA (clone HNK-1), were used to determine NKG2C and CD57 expression on CD56^dim^ NK cells. Samples were analyzed on a Becton Dickinson (San Jose, CA, USA) FACSCalibur flow cytometer and acquired data processed with Kaluza software (Beckman Coulter, Brea, CA, USA). If NKG2C expression as measured by flow cytometry was below 1% of NK, sequence specific polymerase chain reaction was done as previously described to identify NKG2C^null^ subjects homozygous for deletion of a 16-kilobase segment on chromosome 12 encoding the NKG2C gene [29]. Data from these subjects were excluded from our analysis.

### 2.3. Measurement of Anti-HCMV Antibodies

Antibodies against HCMV were measured in plasma samples by ELISA against CMV AD169-infected MRC-5 cell lysate. To generate lysate, 1 × 10^7^ MRC-5 cells were infected with CMV AD169 at a multiplicity of infection of 0.5 and after 3 days were harvested by scraping, pelleted by centrifugation, and lysed in 1 mL lysis buffer. Lysate diluted 1/1000 in carbonate/bicarbonate coating buffer was added in 100 *μ*L overnight at 4°C to wells of Immulon-2 ELISA plates (VWR Scientific, Mississauga, ON, Canada). Lysate prepared as above from uninfected MRC-5 cells was used as control. Plasma samples diluted 1/500 were incubated on the plates for 90 min, washed, and developed with goat-anti-human IgG-horseradish peroxidase conjugate (Jackson ImmunoResearch Labs, West Grove PA, USA) followed by tetramethylbenzidine substrate (Sigma-Aldrich). Colour development ran for 30 min at room temperature, after which the reaction was stopped with 1 N H_2_SO_4_ and optical density (OD) read at 450 nm. CMV AD169 was obtained through the NIH AIDS Reagent Program, AIDS Program, NIAID, and NIH from Dr. Karen Biron [30]. MRC-5 cells were a kind gift from Dr. K. Hirasawa, Division of BioMedical Sciences, Faculty of Medicine, Memorial University of Newfoundland. The in-house ELSA was validated using samples diluted 1 : 100 and tested against recombinant CMV pp150 and gB proteins (Virogen, Watertown, MA, USA) coated on ELISA plates at 50 ng/well. Plasma from samples with the lowest and highest ODs against CMV pp150 and gB were used to define a lysate coating concentration and plasma dilution producing the broadest signal range for the 2 samples over a short incubation period. Then plasma samples from 12 HIV-infected individuals and 12 uninfected controls with no detectable T cell response against either CMV pp65 or IE-1 and OD < 0.10 against CMV pp150 or gB were tested by ELISA against the lysate as described above. For the HIV-infected and uninfected individuals, respectively, mean OD ± standard deviation was 0.035 ± 0.012 and 0.033 ± 0.017. Therefore, we set an OD of >0.10 as conservative threshold for CMV seropositivity by this assay. Subjects with an OD between 0.10 and 0.20 and no detectable T cell response against CMV pp65 or IE-1 are not assigned as CMV-seronegative or seropositive. Only 1 of over 400 subjects tested falls into this category. On each plate, a positive sample initially producing an OD = 2.27 and negative sample with an OD = 0.034 are run as controls and the assay is repeated if there is more than 10% variation in the OD of the positive control or >50% variation in OD of the negative control.

### 2.4. Characterization of HCMV-Specific CD8^+^ T Cell Responses

To enumerate and phenotype both CMV-specific and bystander CD8^+^ T cells, PBMC were thawed, cultured overnight, then counted, and resuspended in 3 aliquots of 2 × 10^6^, each in 1 mL lymphocyte medium. One aliquot served as a negative control, while overlapping peptides from HCMV pp65 or HCMV immediate early- (IE-) 1 proteins (Miltenyi Biotec) were added to the other aliquots in 10 *μ*L RPMI 1640 to yield final individual peptide concentrations of 0.5 *μ*g/mL. The PBMC with peptides were placed in a 5% CO_2_ 37°C humidity-controlled incubator for 1 hour, after which brefeldin A (Sigma-Aldrich) was added to 10 *μ*g/mL and incubation continued for another 4 hr. Following this, the cells were centrifuged at 400 ×g, washed with flow buffer (phosphate buffered saline [PBS] with 5 mM ethylenediaminetetraacetic acid and 0.1% bovine serum albumin [BSA, Sigma-Aldrich]), and surface stained with PerCP^*∗*^anti-CD8 (clone HIT8a, Biolegend, San Diego, CA, USA), FITC^*∗*^anti-CD28 (clone CD28.2, Biolegend), and PE^*∗*^anti-CD57 (clone TB01, eBioscience). The cells were then fixed, permeabilized, and stained with APC^*∗*^anti-interferon-gamma (IFN-*γ*) from eBioscience (clone 4S.B3) or appropriate isotype controls. The processed cells were analyzed on a Becton Dickinson FacsCalibur flow cytometer with acquisition of ≥2 × 10^5^ events/sample.

### 2.5. Statistical Analysis

Data for the groups were represented as medians with interquartile range as the distribution of values measured in most groups was not normal as determined by at least one of the Kolmogorov-Smirnov, D'Agostino-Pearson or Shapiro-Wilk normality tests. Medians were compared between groups by two-tailed Mann-Whitney tests. Spearman correlations were calculated and line of best fit was visualized by linear regression when a significant correlation was observed. Probability values < 0.05 were considered significant. All statistical analyses were carried out with the Graphpad Prism software program, version 5.

## 3. Results

### 3.1. Distribution of CD57^+^NKG2C^+^ NK Frequencies in Different Groups

**Figure 1:**
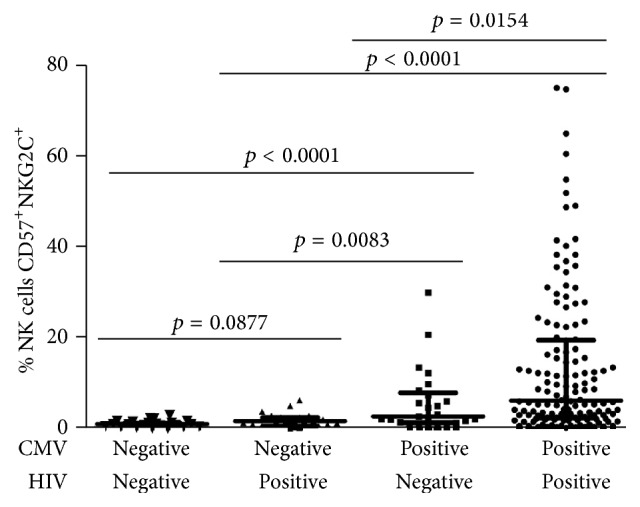
Distribution of CD57^+^NKG2C^+^ NK frequencies in different groups. Flow cytometry was done on PBMC to assess CD57^+^NKG2C^+^ NK frequency by gating on lymphocytes, excluding CD3^+^ cells, gating on CD56^dim^ cells, and plotting NKG2C versus CD57 expression. Horizontal lines bisecting groups represent medians with IQR shown above and below them. Significant differences between medians are shown above lines spanning the groups compared.

The percentage of NK cells expressing CD57 and NKG2C was measured in PBMC from 211 individuals in four different groups distinguished by HIV and HCMV infection status. Age and sex distribution within each group is shown in Table 1. The group of CMV-seropositive, HIV-infected individuals had the highest percentage of CD57^+^NKG2C^+^ NK (median with interquartile range [IQR] = 5.90, 2.30–19.10) followed sequentially by CMV-seropositive controls (2.60, 1.25–7.70), the CMV-seronegative, HIV-infected group (1.55, 0.44–2.30), and CMV-seronegative controls (0.84, 0.34–1.45, Figure 1). There was no significant difference in median age between any of the groups. In the CMV-seronegative, non-HIV-infected group, which had the lowest percentage of CD57^+^NKG2C^+^ NK, there was a significant correlation between age and percentage of CD57^+^NKG2C^+^ NK (correlation coefficient [*r*] = 0.468, *p* = 0.028). No significant correlation between age and percentage of CD57^+^NKG2C^+^ NK was observed for any of the other groups. These data indicate that NK responses driven by CMV are exaggerated in HIV infection, as previously reported for CD8^+^ T cell and antibody responses against CMV [27, 28] and that, in CMV- and HIV-infected groups, factors other than aging have a dominant influence on the extent to which the CD57^+^NKG2C^+^ NK population expands. The median frequency of CD57^+^NKG2C^+^ NK was not significantly higher in the CMV-seronegative HIV-infected group than the CMV-seronegative control group (*p* = 0.0877), indicating that, under most circumstances, HIV infection alone does not promote significant expansion of CD57^+^NKG2C^+^ NK in the absence of HCMV infection (Figure 1).

### 3.2. Relationship between CD57^+^NKG2C^+^ NK Frequency and Antibodies against CMV

**Figure 2:**
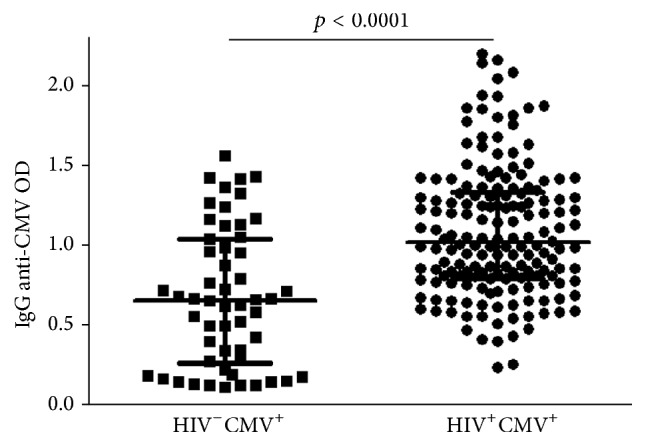
Relative antibody levels against CMV in the HIV-infected and non-HIV-infected group. Plasma anti-CMV antibodies were detected against cell lysate from CMV AD169-infected MRC-5 cells as described in the methods and OD values of the groups shown. Horizontal lines bisecting the groups represent medians with IQR shown above and below them. Significant difference between the medians is shown above a line spanning the groups.

To assess whether NK responses related to antibody responses against HCMV, we next measured relative levels of anti-CMV antibodies in plasma from each CMV-seropositive subject. ELISA OD values ranged from 0.00 to 2.20 following background subtraction and values <0.10 were considered to indicate seronegative status for HCMV. Antibody levels were significantly higher in the HIV coinfected group than in the CMV-infected control group (median with IQR = 1.02, 0.79–1.33 versus 0.65, 0.26 to 1.04, *p* < 0.0001, Figure 2). We tested for significant relationships between age, NK responses, and antibody responses against CMV in HIV-infected and control CMV-seropositive groups by Spearman correlation and found no significant correlations in either group. These data indicate that humoral immunity against CMV develops independently of NK responses against CMV and that factors other than aging have a dominant influence on the size of the response.

### 3.3. Relationship between CD57^+^NKG2C^+^ NK Frequency and CD8^+^ T Cell Responses against CMV

**Figure 3:**
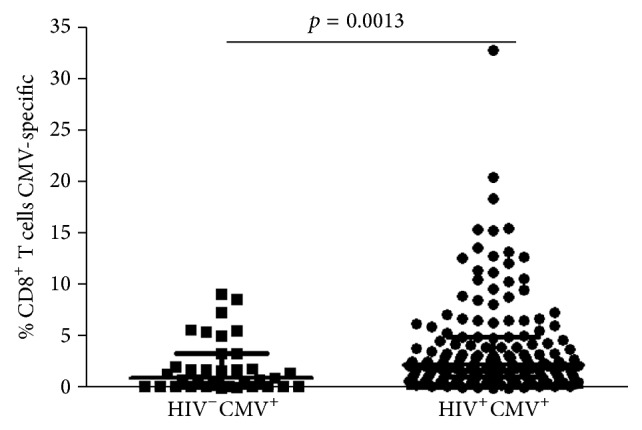
CD8^+^ T cell responses against CMV pp65 and IE-1 in HIV-infected and non-HIV-infected CMV-seropositive subjects. Horizontal lines bisecting the groups represent medians with IQR above and below them. Significant difference between medians is shown above a line spanning the groups.

**Figure 4:**
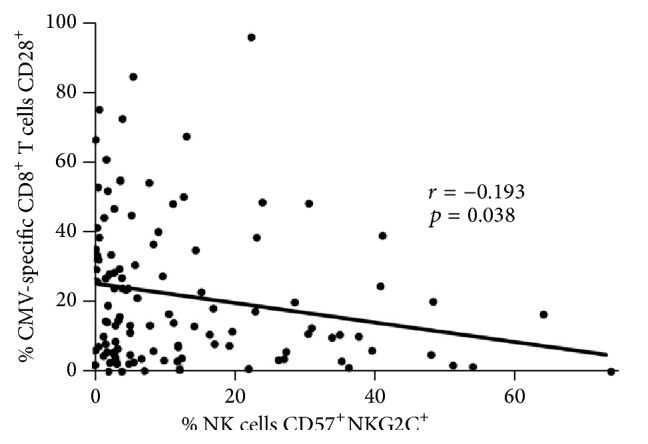
Inverse correlation between CD57^+^NKG2C^+^ NK frequency and the percentage of CMV-specific CD8^+^ T cells expressing CD28 in HIV-infected subjects. In the HIV-infected subject group only, Spearman nonparametric correlation indicated significant inverse correlation between CD57^+^NKG2C^+^ NK frequency and percentage of CMV-specific CD8^+^ T cells expressing CD28. Linear regression was done to produce the line of best fit. The correlation coefficient (*r*) and probability of significant correlation (*p*) are shown within the graph frame.

To assess whether NK responses related to development or evolution of CD8^+^ T cell responses against CMV, we next measured the frequency of CD8^+^ T cells producing interferon-gamma (IFN-*γ*) in response to overlapping peptides from CMV IE-1 and pp65 proteins. We also characterized both the HCMV-specific and bystander CD8^+^ T cell population for surface markers signifying T cell proliferative reserve (CD28) or senescence (CD57). As with expansion of the NKG2C^+^CD57^+^ NK population and anti-CMV antibody levels, median magnitude of the CMV-specific CD8^+^ T cell response was significantly greater in the HIV coinfected group than in the CMV-infected control group (median with IQR = 2.30, 1.00–5.00 versus 1.00, 0.20–3.40, Figure 3). While there was no significant direct correlation between CD8^+^ T cell and NK response magnitudes against HCMV in either group, in the HIV-infected group there was a significant inverse correlation between the percentage of NKG2C^+^CD57^+^ NK and fraction of HCMV-specific CD8^+^ T cells expressing CD28 (*r* = −0.193, *p* = 0.038, Figure 4). As the CD28^+^ fraction of HCMV-specific CD8^+^ T cells also correlated inversely with the magnitude of the CD8^+^ T cell response (*r* = −0.229, *p* = 0.013), this suggests some overlap between features driving HCMV-specific CD8^+^ T cell and NK memory inflation.

## 4. Discussion

To address the impact of innate immune responses against HCMV on development of adaptive immunity against HCMV, we investigated relationships between expansion of NKG2C^+^CD57^+^ NK and the strength or character of adaptive immune responses against HCMV. Since adaptive immune responses against HCMV are often amplified in HIV infection, we included a large group of HIV-infected individuals in order to encompass a range of response strengths as broad as possible [27, 28]. Similar to adaptive immune responses, the innate immune response against HCMV, as indicated by expansion of NKG2C^+^CD57^+^ NK, was exaggerated in HIV infection, reaching levels >70% of all circulating NK. We found no significant relationship between levels of antibodies against HCMV and NKG2C^+^CD57^+^ NK expansion, suggesting largely independent evolution of these responses. Only in the CMV-seronegative control group with the lowest frequencies of NKG2C^+^CD57^+^ NK there was a significant correlation between NKG2C^+^CD57^+^ NK expansion and age, indicating that features other than age or duration of HCMV infection have a dominant effect on expansion of the NKG2C^+^CD57^+^ NK population. In fact, despite the significant correlation, aging in the absence of HIV or CMV infection had only a very minor effect on NKG2C^+^CD57^+^ NK expansion. We did observe a significant inverse correlation between NKG2C^+^CD57^+^ NK expansion and the fraction of HCMV-specific CD8^+^ T cells expressing CD28. Loss of CD28 expression on antigen-specific memory T cells is associated with shortened telomeres, reduced proliferative potential, and progress towards senescence [31, 32]. As this occurs in parallel with NKG2C^+^CD57^+^ NK expansion, it raises the possibility that similar immunological or virological features prominent in HCMV/HIV coinfection drive both NKG2C^+^CD57^+^ NK expansion and HCMV-specific CD8^+^ T cell proliferation underlying memory inflation and senescence. Expansion of NKG2C^+^CD57^+^ NK has been observed in primary HCMV infection and in HCMV reactivation following transplantation, suggesting that active HCMV replication is a major factor at least in initiation of the expansion [20, 33]. Periodic reactivation of HCMV is also thought to drive CD8^+^ T cell memory inflation, and although overt HCMV reactivation with viremia is rare in well treated HIV infection, it is plausible that HCMV reactivation occurs more frequently in HIV-infected individuals. In fact, HCMV shedding in semen and saliva is relatively common in HIV-infected men who have sex with men [34–36]. The parallel evolution of NK and CD8^+^ T cell responses evoked by CMV in HIV-infected persons contrasts somewhat with a report describing general changes in the NK population and T cell repertoire of elderly individuals. In this study, expansion of the NKG2C^+^CD57^+^ NK population was independent of skewing of the CD4^+^/CD8^+^ T cell ratio towards a CMV-related immune risk profile [37]. Another study reported that when CMV replication was well controlled by the immune system, as indicated by low titres of CMV-specific IgG, cellular responses against CMV were dominated by either CD8^+^ T cells or NKG2C^+^ NK, but not both [38]. This suggests compartmentation of NK and T cell responses against CMV, with a reciprocal relationship between their magnitudes. Although we did not observe such a relationship, it may be more likely to occur in individuals who bring CMV under effective and stable immune control quickly and less likely to occur against the background of immune dysregulation associated with chronic HIV infection or unhealthy aging.

The lack of any apparent direct relationships between NK and adaptive immune responses against HCMV observed in our study, despite their common trigger and shared responsibility for containing HCMV, likely reflects a complex, evolving relationship between chronic infection and the multiple immune effector arms raised against it. An inverse relationship between the magnitude of NK and adaptive immune responses against CMV should exist if either class of response alone was sufficient to effectively resolve primary infection and control CMV reactivation. However, since HCMV infection always persists and may continuously reactivate, all arms of the immune response may receive periodic stimulation with unequal reinforcement dependent upon the site and extent of reactivation and concurrent capacities of relevant immune system components to receive and deliver reinforcement signals. Over time, what began as a reciprocal relationship might, therefore, evolve towards a direct relationship or vice versa. We also speculated that a direct relationship between anti-CMV antibody levels and expansion of NKG2C^+^CD57^+^ NK might exist if activation and expansion of this NK subset, specialized to mediate ADCC, was dependent upon interactions with antibody-coated target cells [23–25]. The cumulative impact of inverse or direct relationships operating under different conditions and at different stages of evolution might obscure relevant shorter-term impacts of direct or reciprocal influences in retrospective cross-sectional studies such as this one. Therefore, longitudinal studies beginning in primary infection and investigating responses through primary and chronic stages of infection might be more revealing as to the coevolutionary nature of adaptive and innate immunity against HCMV.

One longer term relationship between innate and adaptive immunity not overshadowed by cumulative effects was an inverse relationship between the extent of NKG2C^+^CD57^+^ NK expansion and the fraction of HCMV-specific CD8^+^ T cells expressing CD28 in HIV infection. In fact, this relationship may represent the common cumulative effect of chronic HCMV infection and periodic reactivation on the T lymphocytes and NK cells that respond to it, at least in the context of HIV infection. Loss of CD28 expression on T cells reflects shortened telomeres and progression towards senescence, generally thought to be driven by persistent exposure to antigen [39, 40]. It also signifies T cell effector memory status and previous specialization to mediate cytotoxicity against infected host cells. The parallel between expansion of NKG2C^+^CD57^+^ NK, their differentiation towards enhanced ADCC potential, and loss of CD28 on HCMV-specific T cells implies that both reflect increased HCMV reactivation to immunogenic levels and a possibly subtle and slow, but ultimately unrelenting, deterioration of immune containment of CMV replication. Just as failure to contain HCMV in the absence of memory inflation in the CD8^+^ T cell response relates to late stage immune dysfunction in old elderly individuals, extensive HCMV-driven expansion of NKG2C^+^CD57^+^ NK may be indicative of a level of underlying immune degeneration in HIV infection.

Restriction of NKG2C^+^CD57^+^ NK expansion to HCMV infection continues to raise questions as to a potential role for NKG2C in recognition of HCMV and as to the nature of the NK stimulus delivered in HCMV infection. While there was no statistically significant expansion of NKG2C^+^CD57^+^ NK in the CMV-seronegative, HIV-infected group compared to the CMV-seronegative, non-HIV-infected group, there was a trend towards an increased percentage of NKG2C^+^CD57^+^ NK. One possibility is that some HIV-infected, CMV-seronegative individuals were exposed to HCMV without seroconversion. There was also broad variability within each of the HCMV-infected groups illustrating the impact of secondary factors presumably related to the course of chronic HCMV infection in driving NKG2C^+^CD57^+^ NK expansion. The tempo or extent of periodic reactivation of HCMV is likely a contributing factor, but there was no significant relationship between anti-CMV antibody levels, often considered as a surrogate for exposure to extracellular HCMV and either NKG2C^+^CD57^+^ NK or HCMV-specific CD8^+^ T cell expansion. Intracellular HCMV reactivation without release of new virions may be sufficient to drive both NKG2C^+^CD57^+^ NK and HCMV-specific CD8^+^ T cell expansion in the absence of any boosting of the anti-HCMV antibody response.

## 5. Conclusions

While both adaptive and NK immune responses against HCMV were stronger in HIV infection, we found no significant correlation between the strength of NK and adaptive immune responses against HCMV. Our data do suggest that NKG2C^+^CD57^+^ NK expansion to high levels reflects stress on the ability of the NK response to keep pace with HCMV reactivation or with other factors underlying this expansion. Thus, we expect adaptation in the character of NKG2C^+^CD57^+^ NK similar to that occurring in virus-specific CD8^+^ T cells failing to contain replication. Accumulation of NKG2C^+^CD57^+^ NK may reflect an NK immune response homologous to the inflationary adaptive T cell response signified by accumulation of HCMV-specific effector memory CD8^+^ T cells. Further functional and phenotypic analysis of NKG2C^+^CD57^+^ NK at different levels of expansion in different virological contexts may shed more light on their relationship to chronic HCMV infection and on the biology of NK responses in general.

## Figures and Tables

**Table 1:** Age and sex distribution of study groups.

Group	HIV^−^CMV^−^	HIV^+^CMV^−^	HIV^−^CMV^+^	HIV^+^CMV^+^
*n*	22	26	25	138
Male (%)	13 (59)	18 (69)	11 (44)	107 (78)
Female (%)	9 (41)	8 (31)	14 (56)	31 (22)
Age in years (median with IQR)	46, 33–53	48, 43–53	48, 39–61	49, 45–55
